# DNA methylation landscape of 16 canine somatic tissues by methylation-sensitive restriction enzyme-based next generation sequencing

**DOI:** 10.1038/s41598-021-89279-0

**Published:** 2021-05-11

**Authors:** Jumpei Yamazaki, Yuki Matsumoto, Jaroslav Jelinek, Teita Ishizaki, Shingo Maeda, Kei Watanabe, Genki Ishihara, Junya Yamagishi, Mitsuyoshi Takiguchi

**Affiliations:** 1 Translational Research Unit, Veterinary Teaching Hospital, Graduate School of Veterinary Medicine, Hokkaido University, Sapporo, Japan; 2 One Health Research Center, Hokkaido University, Sapporo, Japan; 3 Research and Development Section, Anicom Specialty Medical Institute Inc., Yokohama, Japan; 4 Coriell Institute for Medical Research, Camden, NJ USA; 5 Laboratory of Comparative Pathology, Graduate School of Veterinary Medicine, Hokkaido University, Sapporo, Japan; 6 Department of Veterinary Clinical Pathobiology, Graduate School of Agricultural and Life Sciences, The University of Tokyo, Tokyo, Japan; 7 Division of Collaboration and Education, Research Center for Zoonosis Control, Hokkaido University, Sapporo, Hokkaido Japan; 8 Laboratory of Veterinary Internal Medicine, Graduate School of Veterinary Medicine, Hokkaido University, Sapporo, Japan

**Keywords:** Epigenetics, DNA methylation, Zoology

## Abstract

DNA methylation plays important functions in gene expression regulation that is involved in individual development and various diseases. DNA methylation has been well studied in human and model organisms, but only limited data exist in companion animals like dog. Using methylation-sensitive restriction enzyme-based next generation sequencing (Canine DREAM), we obtained canine DNA methylation maps of 16 somatic tissues from two dogs. In total, we evaluated 130,861 CpG sites. The majority of CpG sites were either highly methylated (> 70%, 52.5–64.6% of all CpG sites analyzed) or unmethylated (< 30%, 22.5–28.0% of all CpG sites analyzed) which are methylation patterns similar to other species. The overall methylation status of CpG sites across the 32 methylomes were remarkably similar. However, the tissue types were clearly defined by principle component analysis and hierarchical clustering analysis with DNA methylome. We found 6416 CpG sites located closely at promoter region of genes and inverse correlation between DNA methylation and gene expression of these genes. Our study provides basic dataset for DNA methylation profiles in dogs.

## Introduction

DNA methylation is the conversion of cytosine to 5-methylcytosine at cytosine-guanine (CpG) dinucleotides, causing structural change in the interactions between DNA and protein(s). DNA methylation occurring at promoter regions of genes represses gene transcription^1^. DNA methylation at intergenic regions and gene bodies has also gained attention due to its positive association on gene expression as well^2,3^. In addition, global methylation at CpG loci throughout the genome is thought to reflect change in response to the environment, exposure, immune response, and the carcinogenic process^4^. DNA methylation is a widely recognized regulatory mechanism that is indispensable for cellular development, tissue differentiation, inactive X chromosome in female^5^, and in imprinting^6,7^.

Recently, genome-wide analyses of DNA methylation have revealed that a number of genes are unequivocally differentially methylated in a variety of normal cells as well as tumors^8,9^. However, genome-wide DNA methylation patterns have been well studied only in humans and rodents^10–12^. The sequencing and analysis of the dog genome^13^ will greatly accelerate the dog as a biomedical and spontaneous model for many diseases in humans such as tumors^14^. Although global hypomethylation or hypermethylation at single loci were found in dogs^15–18^, these studies did not search for changes in genomic location modified by DNA methylation. Few genome-wide DNA methylation studies have been conducted for the purpose of identifying differentially methylated CpG sites in diffuse large B-cell lymphoma in dogs compared to peripheral blood/lymph node from healthy control dogs based on microarray platforms^19,20^, however, comprehensive analysis of DNA methylation status among different normal tissues has not been investigated. Given the fact that understanding of DNA methylation pattern in dogs is limited compared to humans and rodents, we previously established Canine DREAM, which is genome-wide DNA methylation analysis in dogs based on next-generation sequencing of methylation-specific signatures created by sequential digestion with *Sma*I and *Xma*I restriction enzymes^21^. In this study, we aimed to construct genome-wide DNA methylation status in 16 normal cell/tissues in dogs for comprehensive understanding of DNA methylation. This study provides basic information on the dog methylome and a reference data for further study of DNA methylation and comparative animal research.

## Results

### CpG sites analyzed

We studied the DNA methylomes of 32 normal dog tissue samples (16 tissue types) including, lung, heart, stomach, duodenum, ileum, colon, liver, pancreas, adrenal gland, kidney, spleen, muscle, skin (white-colored), oral mucosa (white-colored and black-colored), and bone marrow. A complete list of cell and tissue types are shown in Table 1.

**Table 1:** Tissue types used in this study.

	Samples	Number of reads	Number of CpG sites covered	Average CpG methylation per sample	Percent methylated (≥ 0.7)	Percent unmethylated (≤ 0.3)	Percent intermediately methylated (> 0.3 and < 0.7)
1	Lung 73 week-year-old	45,353,568	181,096	61.5	56.5	24.8	18.7
2	Lung 58 week-year-old	35,466,811	169,334	61.7	56.7	24.6	18.7
3	Heart 73 week-year-old	34,954,413	170,173	61.5	56.8	24.6	18.6
4	Heart 58 week-year-old	31,019,481	166,354	61.1	55.8	24.6	19.5
5	Stomach 73 week-year-old	38,545,997	174,204	64.1	61.1	24.0	14.9
6	Stomach 58 week-year-old	37,724,714	173,994	61.9	57.5	25.3	17.2
7	Duodenum 73 week-year-old	41,399,337	175,666	61.3	55.7	23.8	20.5
8	Duodenum 58 week-year-old	47,420,647	176,964	61.2	57.1	26.6	16.3
9	Ileum 73 week-year-old	24,531,063	156,739	63.0	58.3	23.2	18.5
10	Ileum 58 week-year-old	50,538,113	178,711	62.8	58.9	24.8	16.3
11	Colon 73 week-year-old	37,269,930	166,719	62.2	57.0	23.7	19.3
12	Colon 58 week-year-old	37,292,174	171,064	60.9	54.5	24.1	21.4
13	Liver 73 week-year-old	33,074,566	166,541	61.1	55.7	24.8	19.5
14	Liver 58 week-year-old	41,659,584	173,433	59.4	52.8	25.5	21.8
15	Pancreas 73 week-year-old	35,810,465	165,875	60.1	54.9	27.4	17.7
16	Pancreas 58 week-year-old	39,201,439	171,747	60.1	54.9	26.9	18.3
17	Adrenal gland 73 week-year-old	36,444,210	165,838	60.9	56.0	26.1	17.9
18	Adrenal gland 58 week-year-old	34,960,686	170,611	63.1	59.1	23.3	17.6
19	Kidney 73 week-year-old	33,793,352	170,886	61.2	56.3	26.2	17.5
20	Kidney 58 week-year-old	40,913,656	173,920	60.8	55.6	26.5	17.9
21	Spleen 73 week-year-old	44,270,510	173,463	64.3	60.8	23.0	16.2
22	Spleen 58 week-year-old	31,708,030	163,656	65.1	62.4	22.5	15.1
23	Muscle 73 week-year-old	29,969,294	163,433	59.4	52.9	25.5	21.6
24	Muscle 58 week-year-old	44,497,941	167,724	59.0	52.5	26.1	21.4
25	Skin 73 week-year-old	43,810,934	174,647	59.9	53.6	25.5	20.9
26	Skin 58 week-year-old	35,955,601	167,385	60.3	55.0	26.6	18.4
27	Oral 73 week-year-old	34,782,022	164,878	60.6	56.6	28.0	15.5
28	Oral 58 week-year-old	38,799,946	168,123	61.5	56.6	24.9	18.6
29	Oral (pigmented) 73 week-year-old	34,654,969	169,574	59.5	53.6	26.4	20.0
30	Oral (pigmented) 58 week-year-old	48,856,203	178,170	60.2	54.9	26.8	18.3
31	Bone marrow 73 week-year-old	34,852,223	176,838	64.2	64.5	23.4	12.1
32	Bone marrow 58 week-year-old	37,418,398	168,215	64.5	64.6	23.6	11.9

To characterize DNA methylation patterns, we employed Canine DREAM for all of 32 samples. From all 32 samples, 24.5–50.5 million unique usable sequencing reads after conservative filtering (quality filtered and aligned to the dog genome) were successfully generated for DNA methylation analyses (Table 1). We used CpG sites that had more than 20 reads (157–181 thousands CpG sites per sample) to assure quantitative ability. For all the analyses, only autosomal CpGs were considered, resulting in 130,861 common CpG sites obtained for inter-sample comparisons.

**Figure 1:**
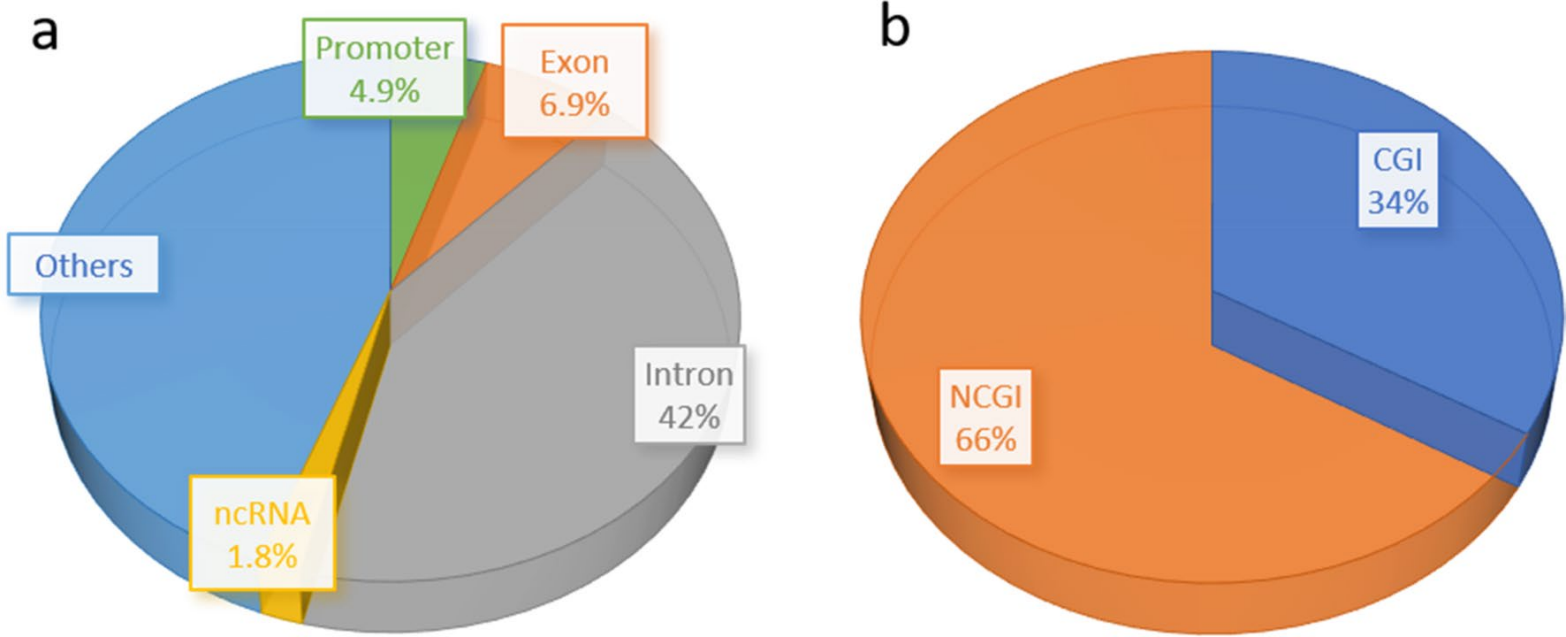
Percentage of CpG sites analyzed in this study in each category of (**a**) genomic features and (**b**) CpG islands.

Of 130,861 CpG sites, 6416 (4.9%) sites were located closely at a promoter region of genes annotated by Ensembl Gene Predictions—version 99. 9002 (6.9%) and 55,065 (42%) sites were located at exons and introns of the genes, respectively. The remaining 60,378 CpG sites were distant from any of the above gene annotations except for 2388 sites located on non-coding RNA such as lncRNA (Fig. 1a). Clusters of CpG sites called CpG islands (CGI) have been recognized to be one of the most important methylation features of the genome and methylated differently from non-CpG island (NCGI) regions in mammals^22^. In this regard, 44,023 sites were in CGIs and 86,838 sites in NCGIs (Fig. 1b).

### Overall status of DNA methylation in each tissue

**Figure 2:**
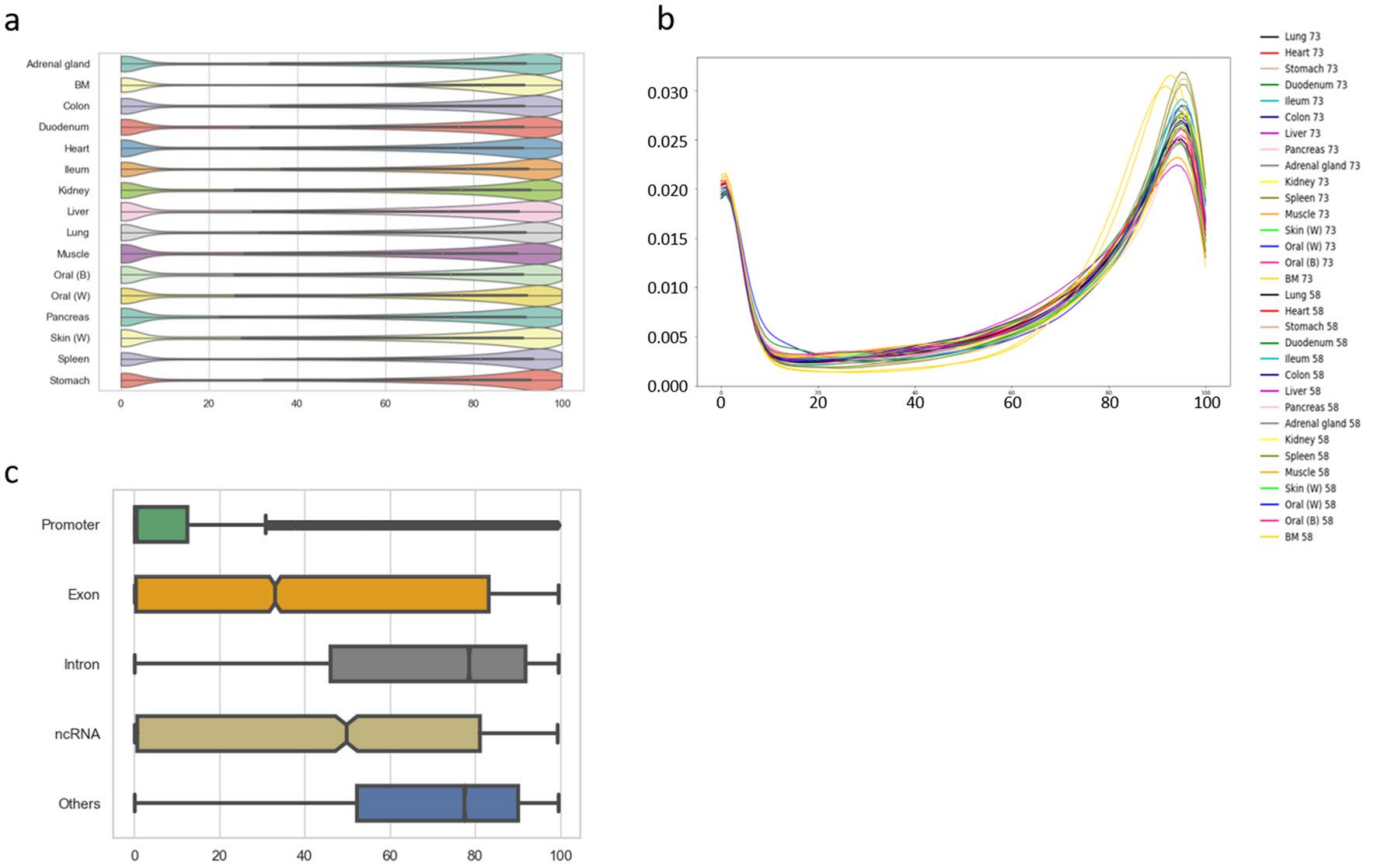
Characterization of DNA methylation patterns across cell types. (**a**) Violin plots of genome-wide DNA methylation levels of all CpG sites for each of the 16 methylome after averaging values for the same tissues. (**b**) Density plots of genome-wide DNA methylation levels of all CpG sites for each of the 32 methylomes. (**c**) DNA methylation level of CpG sites classified by the genomic feature. DNA methylation levels of 6416, 9002, 55,065, 2388, and 57,990 CpG sites at promoters, exons, introns, ncRNA, and other regions, respectively, were calculated first, then averaged for all cell types used in this study. The boxes signify upper and lower quartiles while the median is represented by vertical lines within the boxes. Whiskers indicate maximum and minimum values except for outliers that are shown outside of whiskers. All figures are depicted by seaborn (https://doi.org/10.5281/zenodo.592845).

Next, we addressed overall status of DNA methylation level in all tissue types analyzed. The average DNA methylation levels for each tissue type were 59.0–65.1% with small levels of variation among tissue types. Figure 2a,b show DNA methylation fractions and the overall distribution of the DNA methylation levels. We found that the majority of CpG sites were either highly methylated (52.5–64.6% of all CpG sites analyzed) or unmethylated (22.5–28.0% of all CpG sites analyzed), and the remaining 11.9–21.8% were intermediately methylated (Table 1).

Low level DNA methylation is thought to be a signature of gene regulation^23,24^. Therefore, we sought for the potential of each CpG site in the role in gene expression regulation by identifying the lowest DNA methylation level. We found that 53,830 CpG sites (41.1% of CpG sites analyzed) had their lowest DNA methylation level below 30%. Even though roughly 25% of CpG sites were unmethylated (calculated by the average of the percentage of unmethylated CpG sites in all cell types) for each cell type, 41.1% of the CpG sites could be unmethylated to have potential regulatory functions.

We calculated the average methylation levels for different genomic features in all cell types and found relatively lower methylation levels in promoter regions compared to exons, introns, or intergenic regions (Fig. 2c).

### Tissue specificity determined by genome-wide DNA methylation patterns

**Figure 3:**
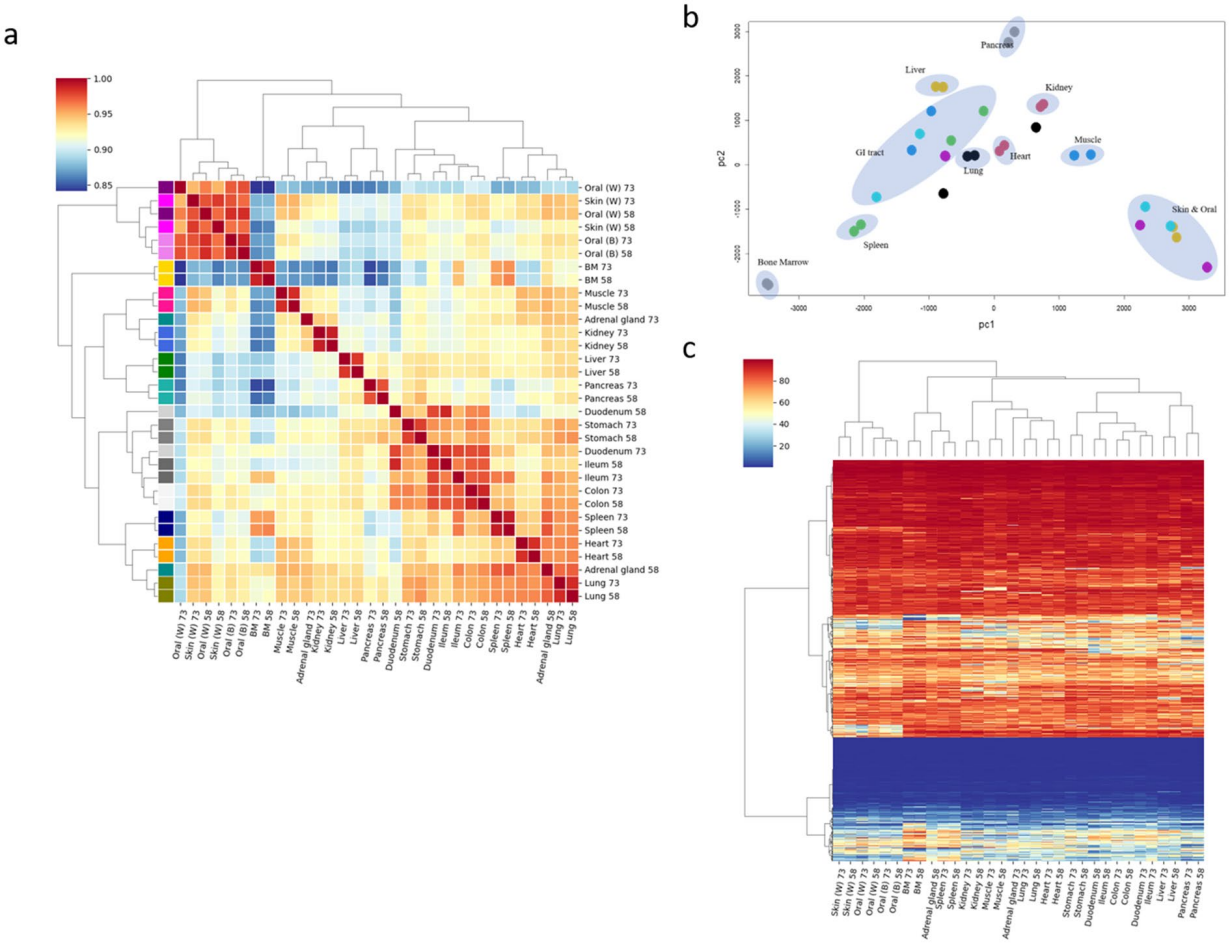
(**a**) Pairplot for correlation scores of DNA methylation levels with all CpG sites analyzed by Canine DREAM. Samples include lung, heart, stomach, duodenum, ileum, colon, liver, pancreas, adrenal gland, kidney, spleen, muscle, skin (white-colored), oral mucosa (white-colored and black-colored), and bone marrow. Also shown is (**b**) Principal Component Analysis (depicted by R, https://www.R-project.org/.) and (**c**) Unsupervised hierarchical analyses of DNA methylation levels with all CpG sites analyzed by Canine DREAM. All figures are depicted by seaborn (https://doi.org/10.5281/zenodo.592845) except for (**b**).

To address if DNA methylome derived from different tissue types could identify tissue specificity, we calculated correlation coefficient for all the pairs (496 pairs) with samples analyzed. As expected, relatively high correlation (R = 0.84–0.99) were observed in all the pairs (Fig. 3a). However, the tissue types were clearly defined by Principal component analysis (Fig. 3b) and hierarchical clustering analysis (Fig. 3c). Interestingly, four gastrointestinal cell type methylomes (duodenum, stomach, ileum, and colon) had notable similarity and so did two epithelial cell type methylomes (oral mucosa and skin).

### Classification of CpG sites by DNA methylation patterns across all samples

The overall distributions of methylated or unmethylated CpG sites from the DNA methylation data of 32 samples were similar, however, genome-wide DNA methylation patterns were variable enough to identify difference of tissue types, indicating that methylation level could be either stable or variable across the samples. To address this hypothesis, we classified CpG sites into constitutively methylated (M), unmethylated (U), and intermediately methylated (I) using 70% and 30% as cutoffs. This resulted in 63,790 (48.7%) of CpG sites either being constitutively methylated (39,116 CpG sites, 29.9%) or unmethylated (24,674 CpG sites, 18.9%). Constitutively intermediately methylated CpG sites were found only in 1.1% (1466 CpG sites) of all CpG sites analyzed.

**Figure 4:**
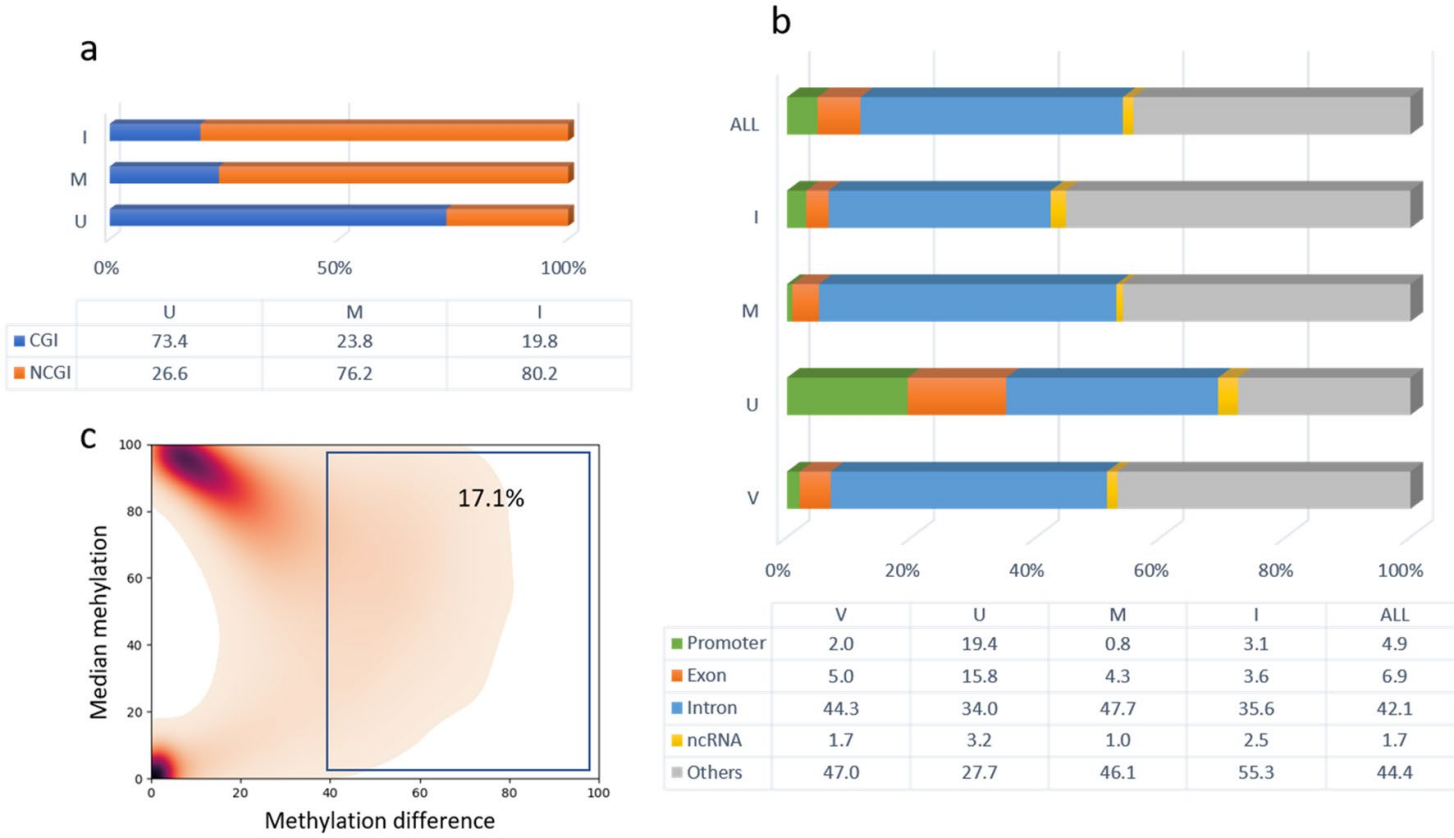
Percentage of constitutively unmethylated (U), methylated (M), and intermediately methylated (I) CpGs analyzed in this study in each category of (**a**) CpG islands and (**b**) genomic features. (**c**) Density scatterplot of CpG-wise DNA methylation level differences (x axis) and CpG median methylation (y axis) across the 32 samples depicted by seaborn (https://doi.org/10.5281/zenodo.592845). Coloring indicates CpG density from low (sparse) to high (dense). The blue box highlights variable CpG sites.

The majority (18,112 CpG sites, 73.4% of 24,674 CpG sites) of constitutively unmethylated CpG sites were in CGIs whereas the majority of constitutively methylated CpG sites (29,800 CpG sites, 76.2% of 39,116 CpG sites) and intermediately methylated CpG sites (1176 CpG sites, 80.2% of 1466 CpG sites) were in NCGIs (Fig. 4a). These findings agreed well with generally appreciated DNA methylation patterns regarding CGI^22^.

Notably, of 24,674 CpG constitutively unmethylated CpG sites, 4777 (19.4%) sites were located closely at promoter region of genes, which was four times higher frequency than that of all CpG sites analyzed. 3898 (15.8%) and 8387 (34.0%) sites were located at exon and intron of genes, respectively (Fig. 4b).

Next, we sought for variable (V) CpG sites across the samples by utilizing definition of having a gap of at least 40% between the third highest and the third lowest methylation values. As a result, 22,385 (17.1%) CpG sites were identified to be variable in our dataset, of which a substantial number of these sites located at intron (9981 CpG sites) or intergenic regions (10,521 CpG sites), leaving only 449 (2.0%) CpG sites located at promoter regions.

### DNA methylation level correlated with gene expression

**Figure 5:**
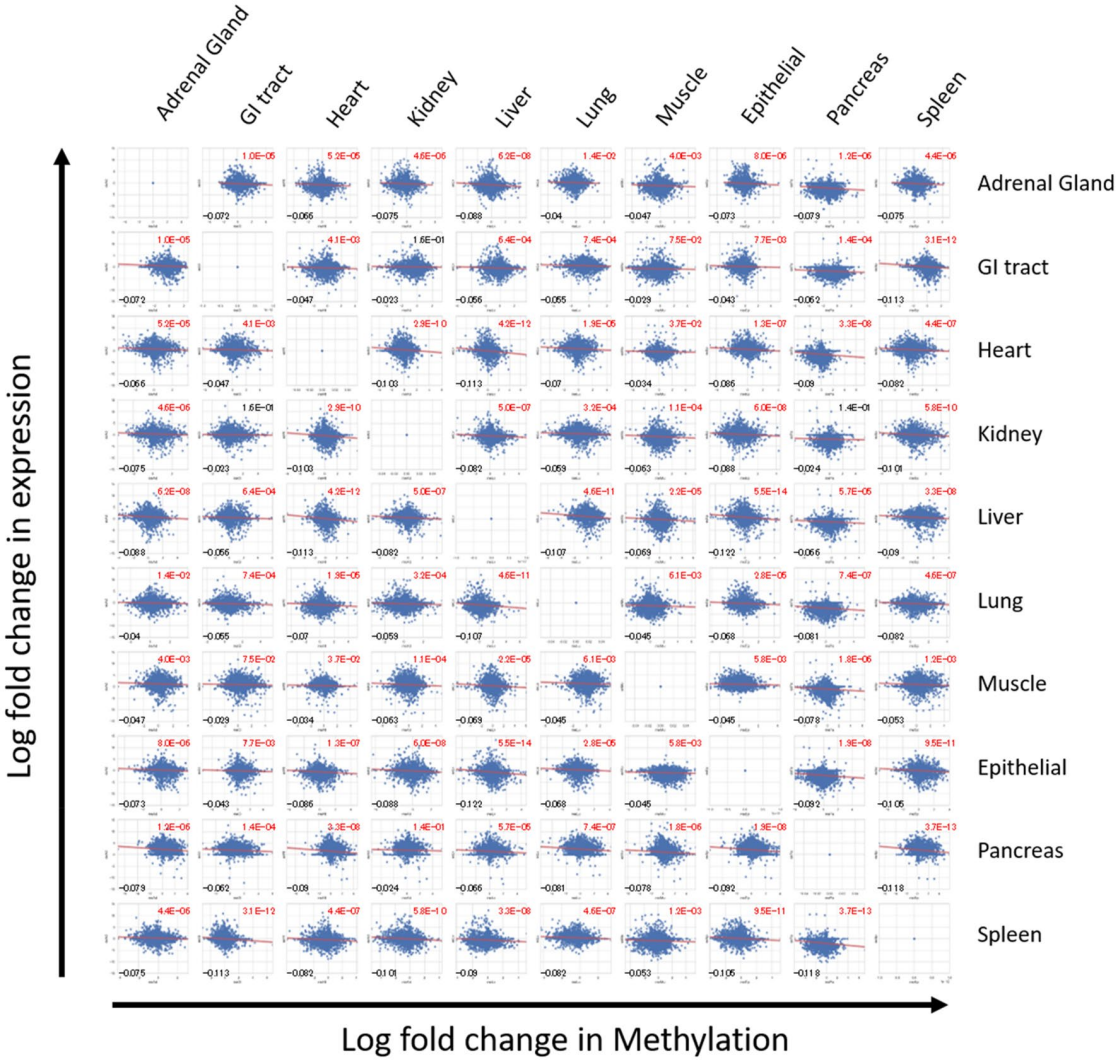
Integration of methylation and expression data. Starburst plot of the 3783 CpG sites that were located at promoter region of transcripts whose gene expression data were available depicted by seaborn (https://doi.org/10.5281/zenodo.592845). Log fold change in DNA methylation (x-axis) and gene expression (y-axis) are plotted for every comparison between each tissue. When the RPKM for a transcript is less than 0.5, the value was adjusted to 0.5. Log fold change were calculated from the value of tissues shown in a row subtracted by tissues in a column. Pearson’s correlation coefficient and p-values were shown in left lower and right upper of each panel, respectively.

DNA methylation at promoter is known to correlates with gene expression silencing^4^. Of 130,861 CpG sites, 6416 (4.9%) sites were located closely at promoter region of genes, which corresponded to 7517 transcripts. We obtained RNA-seq data of the same samples and integrated available gene expression data to observe correlation between differential gene expression and DNA methylation. As a result, we could utilize 3783 CpG sites that were located at promoter region of these transcripts. We plotted differential expression and methylation levels between each pair of all samples after averaging anatomically similar tissues (stomach, duodenum, ileum, and colon were combined intro GI tract. Likewise, skin and oral mucosa samples were combined into Epithelial) (Fig. 5). All of 90 scatterplots showed negative correlation between the two parameters; hypermethylation with gene expression decrease and hypomethylation with gene expression increase between assigned different tissues (r =  − 0.12 to − 0.02, Pearson’s correlation).

Following differentially methylated regions identified in human, the differential methylation has been linked to tissue specific gene expression (Zhang et al. 2013; Lowdon et al. 2014). Therefore, we hypothesized uniquely methylated or unmethylated CpG sites were hallmarks of gene expression in different cell types. In this regard, we extracted CpG sites that were uniquely unmethylated in only one out of 11 tissue types for the following analysis. After the tissues were ranked by their DNA methylation level for individual CpG sites, we defined uniquely unmethylated sites as being only one tissue showing < 30% methylation with a gap of at least 30% between the lowest and the second lowest methylation values. As a result, we identified 1690 uniquely unmethylated sites and found 94 transcripts with these sites at promoter regions. To analyze the relationship between DNA methylation and gene expression for different tissue types, we utilized RNA-seq data for the same samples and found representative 10 transcripts with significantly lower gene expression in the tissue with the second highest DNA methylation levels (Table 2). One of the significantly correlated genes, *PKP3*, was known to play a role in cellular desmosome-dependent adhesion and was found to be highly expressed in epithelial tissue as assumed from the function of the gene.

**Table 2:** Representative uniquely methylated CpG sites with genes.

Chromosome and position	ENSCAFT ID	Gene name	Uniquely methylated tissue	Methylation in uniquely methylated tissue	Expression in uniquely methylated tissue	Second tissue	Methylation in Second tissue	Expression in Second tissue	Expression difference (fold change)	Methylation difference
chr18:25539224	ENSCAFT00000010495.3	PKP3	Epithelial	26.1	316.2	Muscle	78.2	0.5	632.4	52.1
chr7:430493723	ENSCAFT00000027812.4	S100A5	Epithelial	27.7	286.9	GI tract	74.4	64.2	4.5	46.7
chr7:1610651	ENSCAFT00000044289.3	TNNT2	Heart	22.1	1737.2	Epithelial	69.2	3.6	488.5	47.1
chr7:1610651	ENSCAFT00000043795.3	TNNT2	Heart	22.1	20.9	Epithelial	69.2	2.8	7.3	47.1
chr20:37051632	ENSCAFT00000081514.1	ITIH1	Liver	22.8	731.4	Pancreas	80.0	0.5	1462.7	57.2
chr8:68602234	ENSCAFT00000028408.4	SLC25A47	Liver	26.0	479.4	Muscle	72.8	0.5	958.8	46.7
chr10:1130973	ENSCAFT00000000245.4	RDH16	Liver	19.0	410.6	Lung	58.9	36.9	11.1	39.8
chr5:75513913	ENSCAFT00000031965.4	CTRB2	Pancreas	24.2	41,237.5	Spleen	86.2	15.4	2679.7	62.0
chr3:91435257	ENSCAFT00000026438.2	TMED11	Pancreas	15.4	138.4	Kidney	77.0	0.5	276.8	61.6
chr14:6569061	ENSCAFT00000082881.1	CPA1	Pancreas	19.4	15.0	Muscle	73.8	0.5	30.0	54.4

## Discussion

We previously reported Canine DREAM, which is a genome-wide DNA methylation analysis of the dog genome^21^. We utilized Canine DREAM in this study for a variety of normal tissues to construct DNA methylation profiles in dogs and provided basic information for improving our understanding. We analyzed 130,861 CpG sites detected in all samples, which is much more than generally reported DNA methylation studies with strategy that targeted genes of interest, suggesting that Canine DREAM can provide a more in-depth view of DNA methylation status than single-locus studies^17,25^. Compared to genome-wide DNA methylation analysis based on a customized or human microarray platforms^19,20^, analyses with next-generation sequencing followed by mapping to the dog genome will provide flexibility to conduct DNA methylation studies without concern of interspecies difference.

The average DNA methylation levels of all the CpG sites as well as overall distribution of the DNA methylation levels analyzed for each tissue type were quite similar with small levels of variation among tissue types. These findings in the dog were consistent with other species. In this study, the 16 dog tissues showed similar global methylation with correlation coefficients ranging from 0.84 to 0.99. Previously, ten bovine tissues showed correlations ranging from 0.93 to 0.98^26^. The pig study with closely related tissues yielded slightly higher correlations (> 0.95)^27^.

Particularly, 29.9% of CpG sites were constitutively methylated regardless of the cell types whereas 18.9% of CpG sites were constitutively unmethylated. The fact that approximately half of CpG sites are stable in all the 32 samples analyzed in this study is consistent with the fact that DNA methylation is a stable mark across different dog cell types. The majority (73.4%) of constitutively unmethylated CpGs were in CGI, and the majority of constitutively methylated CpG sites (76.2%) and intermediately methylated CpG sites (80.2%) were in NCGIs, which is consistent with the notion that the CGIs were generally less methylated than the NCGIs^22^ and also that methylated NCGIs are suggested to suppress unnecessary retrotransposon expression^28^.

Although overall DNA methylation status among tissue types was similar, principal component analysis and hierarchical clustering analysis with all 32 methylome data showed clear differences indicating tissue-specific DNA methylation patterns. This is consistent with the reports of human and bovine methylome where cell/tissue types were also separated clearly by similar analyses^26,29^. These results suggest that DNA methylation patterns are profoundly involved in tissue differentiation across species. In any given cell type, approximately the same percentage of CpG sites were found methylated and unmethylated, however, methylation status of CpG sites in a certain cell type could be cell-type dependent.

Since our findings indicated different DNA methylome depending on tissue types, we sought for tissue-specific DNA methylation that were associated with gene expression regulation. DNA methylation in promoter region of genes is associated with gene silencing^30^. This was supported by our results that DNA methylation in the promoter regions showed largely negative correlation with gene expression. In addition, we filtered uniquely unmethylated CpG sites at promoter regions in only one tissue type to identify exclusive expression characteristics. As a result, 0 (Adrenal gland)–434 (Pancreas) uniquely methylated CpG sites were found and this variable numbers of uniquely unmethylated CpG sites could partly be attributed to the tissue characteristics in this study.

Widespread colocalization between transcription factor binding and variably methylated CpG sites outside promoter regions were reported in humans^24,29^. Although 22,385 variably methylated CpG sites were found in this study, the majority (> 91%) of these sites located at intron or intergenic regions and could not be analyzed systematically in this regard due to lack of these data in the dog genome. It is no wonder that those variably methylated CpG sites that did overlap with nothing on currently available database might harbor roles in regulation of transcription factor binding which would be identified by increasing amount of transcription factor and histone modification ChIP-seq data in the future.

We expect that more variably and uniquely methylated CpG sites will be identified if more cell types are sequenced and analyzed in the future. We would clearly suggest that DNA methylation plays a role in the regulation of cell or tissue type-specific gene functions. The data and results provided in this study will be useful in the research field of veterinary medicine as well as human medicine. Our methylation data would also be helpful in interpreting the epigenomic status in a variety of cells and conditions such as tumors. It is important to remind that Canine DREAM only analyzed a small part of the dog genome, and more extensive studies such as whole genome bisulfite sequencing are needed to confirm these findings. Nevertheless, our data and many more single-CpG-resolution DNA methylome data available in the future will provide greater insights into the knowledge of epigenetics research field.

## Methods

### Dogs

Two female Beagles 58-week and 73-week-old were used as healthy controls. These dogs were healthy, had no clinical signs, no abnormalities in urinalysis, hematological examination, or blood biochemical analysis. No parasites or pathogenic bacteria were detected in fecal samples. Food was withheld from each dog for 12 h. Dogs then were euthanized and 16 tissues including, lung, heart, stomach, duodenum, ileum, colon, liver, pancreas, adrenal gland, kidney, spleen, muscle, skin, oral, oral (pigmented), and bone marrow were obtained. The use of dogs in this study was approved by the Animal Care Committee of the University of Tokyo (Approval No. P17-064), and the protocol was carried out in compliance with the ARRIVE guidelines and the American Veterinary Medical Association (AVMA) Guidelines for the Euthanasia of Animals (2020). All specimens for Canine DREAM was stored at − 80 °C.

### Digital restriction enzyme analysis of methylation (DREAM)

Genome-wide DNA methylation analysis using next-generation sequencing was performed as previously^21^ with genomic DNA (2 μg) extracted from the above samples. We used the University of California, Santa Cruz (UCSC) definition of CpG islands^31^. Promoter regions are defined as being located within 1 kb from transcription start sites of given genes. Autosomal CpG sites were initially grouped into four biologically motivated categories based on their distribution of DNA methylation values across the 32 samples: constitutively unmethylated (U) if all values were below 30%, intermediately methylated (I) if at least 30 libraries had values between 30 and 70%, constitutively methylated (M) if all values were above 70%.

### RNA-Seq analysis

Total RNA was extracted by the NucleoSpin RNA (Macherey–Nagel, Duren, Germany) for each tissue. RNA integrity number of all RNA is confirmed to be more than 7.0. The RNA-Seq library was constructed by SMART-Seq v4 (Takara Bio) and Nextera XT DNA Library Kit (Illumina). Sequencing was performed with Illumina NovaSeq 6000 according to the manufacture’s software. Quality filtering for sequence reads were performed using Trim Galore (version 0.6.4) with the Phred cutoff score of 30. We used 32–42 million reads after quality control of sequenced reads. Transcripts per million (TPM) was calculated to evaluate gene expression by using Kalisto (version 0.46.2) with default settings and dog gene annotation data sets (CanFam3.1).

### Data analysis and visualization

Violin plots, density plots, boxplots, and Starburst plots were drawn and visualized with Seaborn on Python 3.7^32^. Hierarchical clustering analysis was performed with the agglomeration method ‘ward’ where the distance was calculated with the Euclidean and visualized with Seaborn on Python 3.7. Principal component analysis was performed using R with a package ggfortify 0.4.10^33^. Pearson’s correlation coefficient was calculated for associations between DNA methylation and gene expression levels.

### Institutional animal care and use committee (IACUC) or other approval declaration

This study was approved by the Animal ethical committee of The University of Tokyo.

### Human ethics approval declaration

Authors declare human ethics approval was not needed for this study.

## Data Availability

The dataset generated and/or analysed during the current study are underway to be submitted to Gene Expression Omnibus (GEO) and will be available once it is accepted.

## Abbreviations

CGI: CpG islands
DREAM: Digital restriction enzyme of DNA methylation analysis
